# Mr. Bradley, on Mr. Bellot's Case of Crural Hernia

**Published:** 1808-12

**Authors:** John Alder Bradley


### *To the Editors of the Medical and Physical Journal.*

Gentlemen,

By the early insertion of the following farther Remarks upon Mr. Bellot's Statements, you will again oblige me. There is an error which has crept into my former observations, which my readers will oblige me by correcting with the pen, and more particularly since, though evidently an inadvertency, either in my copy produced by chance, or by an accidental error in the compositor, it has called forth a remark from Mr. Bellot. At page 534, line 31st, from the top, for ' intestine' insert ' omentum.'

---

It is with considerable diffidence I again appear before the Medical World, and more particularly, when the object is to point out what I conceive to be very material errors in practice practice, and mis-statements. Every one who appears before the public claims its attention, and its acquiescence or its disagreement with what he has laid down. If we feel the principles and the practice not to be consonant to the established opinions, we are called upon candidly to state our objections, in order to shew the futility of what is proposed, or to call forth such explanations as may establish the practice brought forwards. Had the operation been in any measure explained by the further statements of Mr. Bellot, I should have acquiesced in silence; but when the practice is adhered to, whilst the objections have not been invalidated, and when new points are furnished, which render the practice more and more objectionable, to come forward becomes a duty.

The points to which the public attention was more particularly drawn, were the following.

1st. As to the possibility of introducing the finger under Poupart's ligament in strangulated crural hernia in the male, so as to separate the connections betwixt the neck of the sac and the contiguous parts.

2dly. As to the possibility of cutting Poupart's ligament "with Pott's bistoury introduced upon the finger, cutting obliquely downwards and towards the os pubis."

3dly. As to the propriety of drawing down the herniary sac, so as to expose its mouth exterior to Poupart's ligament.

4thly. As to the propriety of introducing a bent probe into the sac from above downwards, so as to break down adhesions to the bottom of the tumour.

It is upon these points and the defence that I shall now make some farther comments, and shall likewise shew some statements, as furnished by the reply, to be still more objectionable than those which appear in the original statement.

"The fascia and cellular membrane were cautiously divided the whole length of the tumour, which exposed the hernial sac and ligamentum Poupartii, to which the sac was adhering. With some difficulty it was separated so as to admit the finger, and the ligament dilated with Pott's bistoury, introduced upon the finger." Original Statement, p. 46. vol. 18.

"When the stricture is owing to the ligament only, the difficulty of introducing the finger must necessarily be very great; but when the stricture (as it was in this case) is is at the neck of the sac, the finger then may be got under the arch with more facility, cutting gently with the bistoury upon the tip of the finger." Explanation, p. 260, vol. 20.

The contradiction which exists in these two passages is very obvious; we cannot doubt but the finger may be introduced under the crural arch, if we cut with the bistoury gently upon the tip of it. This then may be granted to Mr. Bellot; but so far from this having been done, as appears by the first Statement, the finger was introduced in Poupart's ligament, breaking down the adhesions betwixt the neck of the sac and ligament; and when this was done, then only was the bistoury used. In the second quotation it is allowed, that we can get the finger under the ligament if we dilate it as we advance with the bistoury; and as it appears by the original statement, that the bistoury was not introduced with the finger in the first place, the inference is an obvious one, and this might be sufficient to set this part of the operation at rest; but, for several reasons, I am at a loss to know how we can get the finger under the arch with more facility when the stricture is produced by the neck of the sac. The peritoneum pushes down before it into the crural sheath, a thin fascia extended from the posterior margin of Poupart's ligament to the pubis, covering the crural ring: this, with the fascia, forming the crural sheath, constitutes the fascia propria of Cooper, the immediate envelope of the herniary sac; the fascia propria, as it descends through the crural ring, lies in contact with the posterior. thin foot of Poupart's ligament, with a small portion of the rounded tendon of the ligament; with a portion of the falciform process of the ligamenta lata, and with the ligament of the pubis; parts which form the boundaries of the crural ring; the herniary sac pushing down this fascia, and taking it for its immediate covering, of consequence, lies in complete contact with it. In some cases of old hernia, a little fat is found interposed betwixt this proper fascia and the body of the herniary sac; this however does not take place at the neck; the fascia propria is close to the parietes of the ring, and the protruded peritoneum to the fascia.

The pressure of the * crural ring upon the neck of the sac

---

* This aperture is very generally small when compared with the size of the hernial sac; and being much pressed upon by the posterior edge of the crural arch, it undergoes a slow process of inflammation, which thickens very much the fascia in which it is enclosed. Cooper, pt. 2. p. 14.

sac produces a slow inflammation, the consequence of which is a thickening of the neck; but the crural ring cannot produce this action in the neck of the sac, unless by acting upon it, which it can only do when the parts are absolutely in contact; it is clear then, that whether the stricture exists at the neck of the sac, or is produced by the crural ring, the complete contiguity of the parts is the same. When the neck of the sac is the point of stricture, this is produced by a thickening of the neck, and not by any contraction of it, producing a more observable distance between the sac and ring.

Mr. Bellot then continues, "In some instances, the arch has been found remarkably wide. Le Dran mentions a case, where one might almost thrust four fingers, with the integuments, underneath it. Obs. de Chirurg. T. 2, Obs. 58." In crural hernia, a dilatation of the ring takes place to a certain extent; this dilatation takes place laterally, the fascia of Gimbernat becomes extended, the crural vessels approach nearer to the anterior inferior spinous process of the ilium, and the consequence is a more close pressure upon the nec of the sac, on its anterior and outer side, by the falciform process of the ligamenta lata, and on its posterior and outer side by the fascia of Gimbernat. If we refer to the plates of Mr. Cooper, this lateral dilatation will be apparent, by comparing the relative situation of the blood vessels as they pass under the ligament and the epigastric artery and vein, with that of the crural ring.† The rarity with which we observe crural hernia attain to any considerable magnitude, when compared with the frequency of the disease, is strong proof of the difficulty with which any of these parts give way; the resistance offered by Gimbernat's fascia, and by the fascia rata, is so very great, that even when the hernia has become of an unusual largeness, we are astonished to see the smallness of its neck; of this, fig. 2 and 3, plate 4th, of Cooper's second part are proofs. This smallness of the neck, even in large hernia, is the strongest possible proof of the great inaptitude to dilatation, and proves, that although large crural hernia do exist, yet the dilatation of the crural ring by no means agrees with the size of the hernia. The largest crural hernia which have been observed by Mr. Cooper, were about the size of the fist, one in the male

---

† Part 2d, plate 3d, fig. 2, with plate 4, fig. 4.

male, the other in the female. Mr. Thompson, of Edinburgh, saw one reaching half way down the thigh,* in a female. Mr. Hey, in a note, page 230, mentions a crural hernia in the female extending to the middle of the thigh. Mr. Lawrence † relates a case of a female, where the sac contained the whole of the jejunum, the ilium, the cœcum, and the ascending colon, with a large share of the omentum; but what makes these three cases not to apply to the question in hand, is, that they all happened in females. It is notorious, that in females the crural hernia always arrives to a larger size than in men; and this is sufficiently accounted for by the greater diameter of the crural ring in females than in males, a circumstance explained by the discovery of Dr. Monro, that the posterior thin foot of Poupart's ligament is considerably broader in the male than in the female.‡ Hence it follows, that the crural ring being naturally larger in females, crural hernia enlarge more freely: the great obstacle to an enlargement in males, consists in the firm resistance offered by this extended posterior insertion of Poupart's ligament.§ From a consideration then of the anatomy of the crural ring, and of the manner in which a thickening of the neck of the sac takes place, so as to produce stricture, I cannot easily admit

* Cooper, part 2d. p. 6.

† Treatise, p 238.

‡ The oval space forming the orifice of the crural sheath, is larger in women than in men. The distance from the spine of the ilium to the symphisis pubis is greater, and consequently the crural arch is longer. The third insertion of the external oblique is not so deep in the male as in the female. The psoas and iliacus internus muscles occupy less space in the female than in the male. I have generally found this disease in women who have a very large pelvis, in whom the ilia and pubis project more than usual. Cooper, part 2d. p. 4.

Vide Monro's Treatise on Crural Hernia, p. 51.

§ In all the instances of strangulated femoral hernia, where I have seen the operation, there has never been room to pass more than the very tip of the operator's finger under the stricture, and frequently even this has been impracticable. I have constantly found the same state of parts in the dead subject, except in the remarkable case related above, (a female) and in one instance where the sac actually contained both intestine and omentum. I could not, after removing the protruded parts, pass my fore finger into the opening. Mr. Lawrence's Treatise on Hernia, p. 239.

Suppose we grant for a moment, that in some cases of large hernia in males, the crural ring may become dilated, this would be of no use to Mr. Bellot, since his case was not of any unusual size, and consequently no unusual dilatation could have taken place; at least, no such thing is noticed, either in the original statement or in the explanation.

mit that we can introduce the finger under Poupart's ligament with more facility when the stricture exists at the neck of the sac.

Again, from a consideration of the above circumstances, I am disposed to adhere to the opinion I formerly gave, as to the impossibility of thrusting the finger under Poupart's ligament, to separate adhesions; and this the more particularly, when I recollect that the subject was a male. Again, from considering the connection of the fascia iliaca, and of the fascia lata with Poupart's ligament, and the extended insertion of the posterior thin foot of Poupart's ligament in males, and from examining the neck of the sac in Cooper's plates, it will hardly appear that the crural arch does in males become very wide; nor is this opinion warranted by the state of stricture which is found by all those practically conversant with the operation.*

These observations would be of little weight were we to regard the statement given by Mr. Bellot from Le Dran; but I shall prove that this case has been placed in a wrong point of view; nay, even that the case itself has never been read. From the manner in which this case is quoted, we should infer, that in an existing crural hernia, a case is given by M. le Dran, where the crural arch was dilated, so that three or four fingers with the skin could be thrust under it: we shall be astonished upon examining the case, to find, that when M. le Dran saw the patient, the hernia with its sac had been returned by the taxis into the cavity of the belly, seven days prior, by M. Arnauld, the son; and the patient was dying from a strangulation of the intestine produced by the neck of the sac within the cavity of the belly. We cannot wonder then, that four fingers, avec la peau, should be thrust into a cavity from whence an hernia and its sac had been displaced.†

Mr.

---

* Vide Lawrence, et supra, and p. 30.
C. Bell's Operative Surgery, vol. 1, p. 297.
Hey's Surgery, p. 150.
Monro's Observations, p. 84, and 85.

† That Le Dran had not been consulted by Mr. B. acquits him of any design of wilful perversion; and that it had not, will be clear upon examining the following quotations. "Le Dran mentions a case, where one might almost thrust four fingers with the integuments underneath it. Obs. de Chirug. T. 2. Obs. 58. Vid. Mr. Bellot's Reply, p. 260.

Le Dran found, in the case mentioned above, that the Fallopian ligament had yielded so much, that one might almost thrust four fingers with the integument

Mr. Bellott then proceeds (p. 260, Expl.). "The necessity of dividing the ligaments will appear, in order to separate

---

integuments underneath it. Obs. de Chirug. T. 2. Observ. 58. Vide Dr. Hull, on Crural Hernia.

Page 25, Med. and Phys. Journal, vol. 11. It might be thought indeed that this similarity in the use of words was accidental, had we not another instance to shew that Dr. Hull has been copied without acknowledgment.

" When such a considerable diversity of opinion has prevailed amongst Anatomists and Surgeons, concerning the part of the arch which ought to be divided, the direction in which the division ought to be made, and the manner of executing it." Mr. Bellot's Reply, p. 259.

" They differ, however, very much as to the part of the arch which ought to be divided, the direction in which the division ought to be made, and the manner of executing it." Dr. Hull, Med. and Phys. Journal, vol. 11, p. 57. These shew clearly the source from which the information respecting Le Dran's case had been drawn, and exculpate Mr. Bellot from any suspicion of having wilfully placed the case in a wrong point of view by a partial statement; indeed, Mr. Bellot has been unfortunate in following Dr. Hull so closely in this point, a point in which that learned gentleman has shewn less of that critical acumen than we generally find him displaying. The case in question is a very important one to surgeons; I shall therefore take the liberty of quoting and making a few remarks upon it, retrenching however such statements as do not strictly belong to the case in a scientific point of view.

Le 5 Mars 1726. On vient me prier à une heure apres midi, d'aller voir le Cocher de M. Denis, rue Thibaud-auz-Dez. Y etant arrivé, on me dit que depuis huit jours il souffroit de grandes douleurs dans le ventre qui avoient commencé par une descente à laquelle il étoit sujet, & qu'il tenoit pour l'ordinaire réduite avec un Brayer: qu'on en avoit fait la réduction, vingt quatre heures apres la sortie des parties; qu'il avoit été saigné deux fois, et que malgre la réduction, les douleurs excessives, et un vomissement continuel avoient subsiste.

Mon confrere M. Arnauld le fils, qui avoit fait la réduction de la hernie, voyant subsister les accidens, avoit fait avaler au malade, quinze ou seize onces de vif argent.

J'examinai le malade, a qui je ne trouvai presque plus de poulx, et qui etoit mourant. Il n'y avoit plus de tumeur dans l'aisne; mais en sa place, on y sentoit une espece de vuide & le ligament de Fallope s'etoit tellementt prêté au volume de la hernie, qu'on pouvoit presque fourer, les quatre doigts avec la peau, par dessous.

Je presumai que l'intestin remis dans le ventre avec le sac herniaire y etoit encore enfermé, et étranglé ...... le malade mourut a cinq heures du soir.

L'ouverture du corps vérifia ceque j'avois dit. Nous trouvames dans le ventre le sac herniare, qui avoit trois pouces de profondeur sur huit pouces de circonférence, et dans ce sac etoit encore enfermée une demee aulne de l'intestin jejunum. Tenant le sac a pleine main, je voulus en faire sortir l'intestin, en le tirant par l'un des bouts; mais la chose me fut impossible, tant l'entrée du sac étoit resevrée, et je n'en vins à vout qu'en dilatant cette entrée avec les ciseanx. Comment l'intestin auroit il pû sortie du sac, et rentrer par le taxis? Tout la portion de l'intestin jejunum, au dessus de l'étranglement étoit très dilatée, pleine de liquide, et de vif argent; et par l'inflammation, elle avoit contracté pres d'un travers de doigt d'epaisseur, a toute separate the adhesions round the neck of the sac, and to get above the mouth of it, as no opening could safely be made below from the adhesion which had taken place." In the operation for crural hernia, no one doubts the propriety of dividing some portion of the ligament surrounding the neck of the sac; not, however, for the reasons here given, neither did Mr. Bellot divide it for the reasons stated. The ligament was divided, and an attempt made to get into the sac below the stricture, which failed; then only was it found out that adhesions between the sac and its contents existed (see Original Statement). The ligament could not be divided on account of adhesions, which he did not know at that time existed; and as the opening above the stricture was made in consequence of these adhesions being found, it follows, that neither could the ligament have been divided on this account; and consequently, that the reasons given for the division of the ligament are incorrect. Had we no other reasons but these for dividing the crural ring, how very seldom would it be necessary, and yet we know it to be indispensible in every operation.

We now come to examine the explanation respecting direction of the incision, and I am here told to look at the patient as he lies upon the operating table, in order to

---

a toute sa circonference. Le mesentere meme qui soutient le jejunum avoit par la meme raison contracté une epaisseur sur naturelle, et les vaisseaux qui y repondent, étoient tres goufles et gorges de sang. Vide Observat. de Chirurgie, Tom. 2d. Obs. 58.

In this case an herniary sac, containing half an ell of intestine, was returned into the cavity of the belly, and the patient lived seven days afterwards; upon dissection, the herniary sac was found measuring three inches in depth, and eight in circumference; the contained intestine was full of liquid and quicksilver. Now the quicksilver was given after the return of the herniary sac, which proves that the communication from above with the strangulated portion was not completely interrupted, and consequently, that previous to reduction the fluid contents of the sac might have been pressed into the belly. In this case, a tumour of the size when measured upon dissection, was not passed through the crural ring; the fluid contents had passed into the abdomen, and a knuckle of intestine and the herniary sac where returned. The case was an enterocele. The state of the crural ring may be judged of by the state of the neck of the sac; such was the tightness of the neck, that the intestine could not be drawn away till it was dilated by the scissars: this sufficiently proves the little dilatation the crural ring had undergone. We can only account for M. Le Dran being so much deceived by considering the anatomy of the crural arch; the fingers were introduced into the space from whence the herniary sac had been dislodged, and the partial pressure upon the fingers produced by the falciform process of the ligamenta lata, was mistaken for Poupart's ligament. A case in some respects similar may be found in Cooper, Part 2d. page 26.

allow that we can cut Poupart's ligament in the direction stated. In whatever position the patient may be placed, an incision upwards will still be in the direction of a line drawn from the pubis to the ribs. When a surgeon speaks of an incision upwards or downwards, the expression is guided by a consideration of the patient in a natural and erect position; and the direction of an incision obliquely downwards and towards the pubis, will be clearly expressed by the words. We here find an addition given to the former statement of the manner in which the incision was made, not indeed rendering it more intelligible or more practical. "The ligament was dilated with Pott's bistoury, introduced upon the finger, cutting obliquely downwards and towards the pubis," (original stat.)—"The incision was begun nearly in a direction towards the umbilicus, *(i. e. upwards and inwards)* then turning the edge of the bistoury obliquely towards the pubis, carrying it downwards in order to avoid the spermatic end." (*Spermatic chord, I presume*) Expl. p. 260. The kind of incision as explained, although different, is not less singular than the original one. Upon attentively considering this incision, it will appear, that such an one would absolutely cut away a portion of Poupart's ligament: but I contend that such an incision was not practicable; the first incision through the integuments being only a simple longitudinal one, could not allow room for so hazardous and complex an incision into the ligament; and of this we shall be more convinced, when we attend to the manner in which the crural hernia is tilted up over the crural arch. Upon these accounts I must still beg leave to adhere to my former opinions. A method of cutting Poupart's ligament, diametrically opposite to either of those which are said to have been executed, (for, as above stated, that in the explanation differs from that stated in the original) is next quoted,* and

* Dr. Hull's mode differs very slightly from Mr. Elsis, which, as we are told by Mr. Cooper, Part 2d, p. 18, Mr. Borret put in execution, but with great difficulty passed the director under the crural arch, from an opening made above it into the tendon of the M. obliq. ext. The case to which Mr. Cooper alludes, is most probably the one published in the M. and P. Journal, vol 5, p. 116, by Mr. Borret. Since Dr. Hull's mode appears very feasible, and the quotation is given incorrectly from Dr. Hull's statement in Ellen Livesey's case, where the Dr. attempted to put in execution Mr. Elsis's plan, and was foiled, I shall here give his own words. "A small aperture is to be made through the tendon of the M. obliquus externus, about half an inch above the crural arch, at which the point of a grooved and we are told that this would be a good general rule to follow! May I then have leave to ask, why was it not followed?

Quo teneam vultos, mutantem protea nodo!

I have before said, that points rendering the operation still more objectionable, had been furnished by the defence, the chief to which I allude now comes under review. "After the division of the ligament, and the adhesions were separated, the whole of the sac, with its contents, might have been returned without opening the sac," p. 260. The herniary sac then was dissected up and separated from the surrounding parts: might I be allowed to ask with what object? certainly none; the adhesions betwixt the sac and its contents could not be more easily separated; the sac could not be more easily or safely opened. If the knife is used, the dissection is full of difficulty and danger, on account of the approximation of the large femoral artery and vein; nor could the herniary sac be thus completely separated from its adhesions, by tearing asunder its connections with the fingers. The only objects which could justify so complete and so dangerous a detachment of the sac, are those of cutting it away; returning it into the cavity of the abdomen; or of closing up its mouth by the royal stitch.* None of these were executed, an herniary sac, by a long, a difficult, and a dangerous dissection, was detached from its adhesions; and for what purpose? that it might be laid down again! I know of no author by whom such a proceeding is advised; and I think that the honour of introducing this is justly due to him who put it

L l 2 in grooved director is to be introduced, and being kept in close contact with the posterior surface of the tendon, passed down to the arch; this portion of tendon is then to be divided upon it, by a probe pointed bistoury with a narrow blade. An assistant is now to draw the spermatic chord upwards, with a blunt hook, or bent probe, and the crural arch is to be cautiously divided from before backwards, till it be entirely cut through, or till the crural ring appear to be sufficiently enlarged; and in doing this the edge of the knife should be turned towards that portion of the linea alba, which lies betwixt the umbilicus and symphysis pubis, that the arch may be cut obliquely inwards, near to its insertion in the os pubis." Dr. Hull, M. and P. Journal, vol. xi. page 254.

* Le Dran thinks there would be no difficulty in tying the hernial sac in crural hernia, "dans la hernie crurale, je ne vois aucune difficulte, de faire le ligature du sac." Obs. de Chirurg. Tom. 2, page 21. It would appear very difficult, upon considering the depth of the neck of the sac, "*l'endroit le plus etroit*;" but we ought always to regard with veneration, the practical opinions of so great a man.

in execution. And yet we are told, that every deviation from established rule is on the safe side.

"The adhesions prevented the contents being pushed back, and also from getting into the sac below its neck; when this is the case, Monro recommends the tumour to be dragged down, to get into the sac from above downwards." Exp. 261. Dr. Monro, as I have before stated, advises, when there are adhesions, that we should attempt to get into the sac from above, downwards, through the neck; but no where, that for this purpose the sac should be dragged down. The herniary sac was first dragged down, and then an attempt made to get into it below the stricture; adhesions were found, we are told, and then the attempt was made to get into the sac, by an opening in the peritoneum above its mouth. It is clear then that the motives are mis-stated in the above quotation, as will be evident upon comparing the above with the following. "Cautiously tearing asunder the adhesions betwixt the sac and the ligament all round, I then drew down the tumour a little, which brought to view the mouth of the sac. Here there appeared to be considerable stricture. I several times attempted to raise the sac from its contents, in order by slight scratches to make an opening into it; but this was ineffectual, as a complete adhesion had taken place between the sac and its contents, throughout the whole of the tumour. At length I succeeded in making a small opening a little above the mouth of the sac." Orig. Stat. p. 46 and 47. The elasticity of the peritoneum is next urged as a proof of the *possibility* of dragging down the sac. I deny not the capability of extension of the peritoneum, I only contend, for the reasons I have before stated, that the force necessary to expose the mouth exterior to Poupart's ligament, cannot be exerted with safety to the patient, and more particularly when such a train of symptoms are present, symptoms which Mr. Bellott allows, "*are the forerunners of death, and denote a speedy dissolution.*" (Orig. Statement.)

"This division *(of the ligament)* together with stretching the sac, brought the *neck* into view." (Explan. 261.) "I then drew down the tumour a little, which brought into view the mouth of the sac." (Original Statement, 47.) Upon these I have to remark, that the neck of the sac and the mouth are distinct parts; and that if the neck of the sac only was exposed, as stated in the explanation, then the mouth was not exposed, as stated in the original. The neck of the sac is half an inch and more in length; and and to expose its mouth, it would be necessary to drag down the sac for this extent. We are told that Poupart's ligament being divided, such an extensive dragging down of the sac would not be necessary. Of the division of the ligament my ideas are by this pretty well known; but granting all this, the danger of attempting to pull down the sac will be apparent.

"The possibility of breaking adhesions with success, is evident from the result of the case. (Expl. 261.) My objections have been made to the propriety; the possibility I have granted to the fullest extent, if a surgeon is found hardy enough to risk the force necessary to break down adhesions of such a nature, unseen, and by guess. The obvious mode of prosecuting the operation, at this period, consisted in introducing a bent probe just through the stricture, and upon its *probe* point making a small hole sufficient to push through the point, and afterwards either cutting the neck of the sac upon the probe or a director. (Vide Appendix to Monro on Crural Hernia, page 14) the adhesions would then have been separated, without the risk attending, blindly pushing down a probe to the very bottom of the tumour. To thrust a probe to the bottom of an herniary sac, where the contents adhere to every part of it, and to each other, is certainly a deviation from established rule, and that not upon the safe side. The only thing I shall now bring forward is an assertion advanced rather too hastily, "The opening which was made above the mouth of the sac, was not made into the cavity of the belly." The incorrectness of this is too evident to require a serious refutation.

When the extensive diffusion of this Journal is considered; when we recollect that not only the experienced, capable of forming accurate judgments upon the cases given to the public, but the young and inexperienced, are its readers, it surely cannot be considered intrusion, that I have thus extended my remarks upon so important a subject. The ideas of the latter are two often attracted by the outward shew and brilliancy of an operation; when the young mind dwells upon single ideas or impressions, they are with difficulty eradicated as the judgment becomes more matured. When cases are thus brought forward, in opposition to anatomy and surgery, it is of the greatest importance they should be placed upon their true and proper grounds; and that they should stand or fall by their own intrinsic weight. The leading points of objection have been increased in number by the explanation. If the o-

 peration peration, as thus said to have been executed, keeps its ground, it is evident that a complete revolution will take place so far as goes to the operation for crural hernia; but of this I feel it cannot do. Again, I apologize for the great length of my remarks, and can only plead the necessity induced by the importance of the subject.

From the collision of different sentiments, sparks of truth are often elicited.

I am, &c.

*September* 28, 1808. JOHN ALDER BRADLEY.

*In the midst of a controversy, it might seem partial hastily to refuse a paper sent by an author in his own defence; we reserved however the above without noticing it in our correspondence of the last month, doubtful how to dispose of it. As controversy cannot be entirely avoided in a work the object of which is mutual improvement, we take this opportunity of requesting that such gentlemen as find it necessary to defend themselves, or to offer useful remarks on others, will do it with perfect politeness, and with as much brevity as possible.*

